# A novel lncRNA PLK4 up‐regulated by talazoparib represses hepatocellular carcinoma progression by promoting YAP‐mediated cell senescence

**DOI:** 10.1111/jcmm.15186

**Published:** 2020-04-03

**Authors:** Yan Jia, Huanhuan Jin, Liyuan Gao, Xiang Yang, Feixia Wang, Hai Ding, Anping Chen, Shanzhong Tan, Feng Zhang, Jiangjuan Shao, Shijun Wang, Shizhong Zheng

**Affiliations:** ^1^ Department of Pharmacology School of Pharmacy Nanjing University of Chinese Medicine Nanjing China; ^2^ Department of Pharmacology School of Pharmacy Wannan Medical College Wuhu China; ^3^ Department of Surgery Nanjing Second Hospital Nanjing China; ^4^ Department of Pathology School of Medicine Saint Louis University St Louis MO USA; ^5^ Department of Hepatology Integrated Traditional Chinese and Western Medicine Nanjing Second Hospital Nanjing China; ^6^ Jiangsu Key Laboratory for Pharmacology and Safety Evaluation of Chinese Materia Medica Nanjing University of Chinese Medicine Nanjing China; ^7^ Jiangsu Key Laboratory of Therapeutic Material of Chinese Medicine Nanjing University of Chinese Medicine Nanjing China; ^8^ Shandong co‐innovation Center of TCM Formula College of Traditional Chinese Medicine Shandong University of Traditional Chinese Medicine Jinan China

**Keywords:** cellular senescence, hepatocellular carcinoma, polo‐like kinase 4 associated lncRNA, talazoparib, Yes‐associated protein

## Abstract

A growing number of studies recognize that long non‐coding RNAs (lncRNAs) are essential to mediate multiple tumorigenic processes, including hepatic tumorigenesis. However, the pathological mechanism of lncRNA‐regulated liver cancer cell growth remains poorly understood. In this study, we identified a novel function lncRNA, named polo‐like kinase 4 associated lncRNA (lncRNA PLK4, GenBank Accession No. RP11‐50D9.3), whose expression was dramatically down‐regulated in hepatocellular carcinoma (HCC) tissues and cells. Interestingly, talazoparib, a novel and highly potent poly‐ADP‐ribose polymerase 1/2 (PARP1/2) inhibitor, could increase lncRNA PLK4 expression in HepG2 cells. Importantly, we showed that talazoparib‐induced lncRNA PLK4 could function as a tumour suppressor gene by Yes‐associated protein (YAP) inactivation and induction of cellular senescence to inhibit liver cancer cell viability and growth. In summary, our findings reveal the molecular mechanism of talazoparib‐induced anti‐tumor effect, and suggest a potential clinical use of talazoparib‐targeted lncRNA PLK4/YAP‐dependent cellular senescence for the treatment of HCC.

## 1 INTRODUCTION

Liver cancer is the second common malignancy worldwide.^1^ Primary liver cancer includes hepatocellular carcinoma (HCC) and intrahepatic cholangiocarcinoma (ICC).^2^ HCC accounts for approximately 90% of primary liver cancer incidence.^3^ The tumour invasive growth and metastasis lead to poor prognosis in patients with HCC.^4^ Therefore, it is urgent to reveal novel molecular mechanism in HCC progress and identify specific diagnostic and prognostic markers, which might provide potential therapeutic targets to improve patient survival.

Long non‐coding RNAs (lncRNAs) are greater than 200 nucleotides in length and exhibit limited or no capacity to encode protein. LncRNAs could interact with RNA, DNA and protein, and further mediate various life process, including epigenetic regulation and cell cycle regulation.^5^, ^6^ Recently, studies have reported that some certain lncRNAs specifically exist in differentiated tissues or tumour cells.^7^, ^8^ These lncRNAs play important roles in regulating cancer cell growth and maintaining tumour characteristics.^9^ Attractively, the abnormal expression of lncRNAs involves in hepatocarcinogenesis and metastasis.^10^, ^11^ However, the cellular functions of lncRNAs in HCC occurrence and development, especially potential cross talk between lncRNAs and cancer‐related signalling pathways, remain largely unknown. Our recent work identified a number of up‐regulated and down‐regulated lncRNAs during HCC. Among these, the expression of polo‐like kinase 4 associated lncRNA (lncRNA PLK4), upstream of the PLK4 gene, was most significantly decreased in HCC patients, compared with normal group. We then first demonstrated that poly‐ADP‐ribose polymerase 1/2 (PARP1/2) inhibitor, talazoparib, could increase the expression of lncRNA PLK4 in liver cancer cells. However, whether talazoparib‐induced lncRNA PLK4 could and how to regulate HCC development is unclear and warrant further study.

Cellular senescence, a special form of durable cell cycle arrest, has attracted great attention. Cellular senescence suppresses proliferation in different types of damaged cells and it is usually associated with cancer and aging.^12^ Senescent cells are characterized by DNA damage, increase of senescence‐associated β‐galactosidase (SA‐β‐gal) positive cells and decrease of telomerase activity. The senescence mediators mainly include tumour suppressor p16^INK4a^ (abbreviated as p16), p53 and cyclin‐dependent kinase inhibitors p21 and p27. Besides, chromatin protein Hmga1 and DNA damage maker γ‐H2AX also contribute to senescent phenotypes.^13^, ^14^ Cellular senescence is best known as a cell‐intrinsic death to prevent liver cancer.^15^, ^16^, ^17^ It is interesting to explore whether talazoparib‐induced lncRNA PLK4 mediates HCC cellular senescence and further to explore the regulatory mechanism underlying its functions.

A growing number of studies including ours have established Hippo/Yes‐associated protein (YAP) signalling as a growth‐suppressive pathway.^18^, ^19^, ^20^ In normal body, the level of YAP phosphorylation maintains balance for cytoplasmic sequestration and degradation. Under cancer state, YAP becomes activated and subsequently translocates into the nucleus, interacting with TEA domain (TEAD) transcriptional factors, and consequently promoting transcription of downstream genes, associated with cell proliferation and survival.^19^, ^21^ Recently, YAP has been identified as a central oncogenic regulator for HCC.^22^, ^23^, ^24^ Besides, the regulation between lncRNAs and YAP offers a promising perspective for the treatment of HCC.^25^, ^26^, ^27^ For example, the overexpression of ultra‐conserved lncRNA uc.134 inhibits HCC cell proliferation and invasion by down‐regulating Cullin4A, an important component of multiple cullin‐RING–based E3 ubiquitin‐protein ligase complexes, further to induce the serine/threonine kinase LATS1 ubiquitination and increase phosphorylated YAP expression.^26^ Moreover, numerous studies have reported that the inhibition of cellular senescence was dependent on YAP activation.^28^, ^29^, ^30^ Thus, investigation on whether lncRNA PLK4 involves in YAP‐mediated cellular senescence of HCC cells is interesting.

In our study, lncRNA microarray indicated that lncRNA PLK4 is significantly down‐regulated in HCC patient tumour tissues. We then find that talazoparib could increase the expression of lncRNA PLK4 in HepG2 cells. Besides, we first demonstrate that the up‐regulation of lncRNA PLK4 induced by talazoparib restricts HCC cell proliferation by promoting cellular senescence. Importantly, we confirm that talazoparib‐induced lncRNA PLK4 inhibits the expression of YAP by decreasing its promoter activity to trigger HCC cellular senescence. Taken together, our study shows that talazoparib‐induced lncRNA PLK4‐YAP‐senescence signalling axis could be a molecular basis for HCC therapeutics.

## 2 MATERIALS AND METHODS

### 2.1 Chemicals and reagents

Talazoparib was brought from Selleck. 10% dimethylacetamide (#270555) and 5% Kolliphor HS 15 (#42966) were obtained from Sigma‐Aldrich. Dulbecco's modified essential medium (DMEM), foetal bovine serum (FBS) and Opti‐MEM medium were bought from GIBCO BRL. TERT antibody (ab32020) was purchased from Abcam Technology (Abcam). Antibody against antimouse IgG (SA00001‐1), anti‐rabbit IgG (SA00001‐2), β‐actin (60008‐1‐Ig), γ‐H2AX (10856‐1‐AP), p16 (10883‐1‐AP) and p21 (10355‐1‐AP) were purchased from Proteintech Group, Inc Primary antibodies against Hmga1 (7777), Ki67 (9449) and YAP (14074) were procured by Cell Signaling Technology.

### 2.2 Microarray assay

Fresh paired normal and histologically confirmed liver tumour tissues were gained from HCC patients, getting no treatment during surgery from the Nanjing Second Hospital. The study was approved by the Ethics Committee of Nanjing Second Hospital, and each patient obtained informed consent.

Human lncRNA microarray is analysed for the global profiling of human lncRNAs and protein‐coding mRNA transcripts. A total of 35 620 lncRNAs were examined in the array. The widely accepted public transcriptome databases (RefSeq, UCSC Known Genes, Ensembl, etc) and landmark publications were applied to construct lncRNAs. Agilent GeneSpring GX v12.1 software package was used to conduct quantile normalization and subsequent data processing. The differential lncRNAs with statistical significance were identified using volcano plot filtering. The threshold used to filter up‐regulated or down‐regulated lncRNAs was a fold change ≥2.0 and *P* < .05. The roles of differentially lncRNA‐targeted mRNAs in the corresponding biological pathways or gene ontology (GO) terms were determined using KEGG pathway analysis and GO analysis.

### 2.3 Animal treatment

Experimental protocol obtained the approval of the animal welfare body in Nanjing University of Chinese Medicine (Nanjing, China). All animals were given humane care according to the National Institutes of Health guidelines. Male BALB/c nude mice were bought from Nanjing Medical University (Nanjing, China). After adaptive feeding for one week, 2 × 10^8^ Huh‐7 cells were subcutaneously injected into the left flanks of mice to establish subcutaneous xenograft model. As previously described,^31^ talazoparib was dissolved in 10% dimethylacetamide and 5% Kolliphor HS 15 in PBS and given once daily via intragastrical (i.g) administration for 3 weeks. Mice were anaesthetized by injecting pentobarbital (50 mg/kg) and then sacrificed at the end of the experiment. Heart, liver, spleen, lung, kidney and tumours were fixed in10% formalin for histopathological studies.

### 2.4 Tumorigenesis assays in nude mice

Tumour size was measured twice a week. Tumour volume was assessed using the formula, tumour volume = (length × width ×height)/2.

### 2.5 Histopathology and immunohistochemistry

As previously described, haematoxylin and eosin (H&E) were performed. According to the previous method,^32^ immunohistochemical staining was conducted using antibodies against Ki67.

### 2.6 Cell culture

HepG2, Huh‐7, LX2, LO2 and SMCC‐7721 cell line were brought from Cell Bank of Chinese Academy of Sciences. HepG2, Huh‐7, LX2, LO2 and SMCC‐7721 cells were cultured in high‐glucose DMEM with 10% FBS and 1% antibiotics, and incubated in a humidified atmosphere at 37°C with 5% CO_2_ and 95% air.

### 2.7 Cell viability assay

Cellular viability was determined by Cell Counting Kit‐8 (Beyotime Biotechnology). Briefly, 100 μL HepG2 cells per well were plated into 96‐well plates. After the corresponding reagents treatment, 10 μL CCK8 solution was incubated with cell medium for 2 hours at 37°C. The absorbance of each well was detected at 450 nm by a Microplate Reader (Bio‐Rad).

### 2.8 Cell cycle analysis

Cell cycle analysis was detected by Propidium Iodide Flow Cytometry Kit (Abcam). HepG2 cells were cultured in 6‐well plates and treated with indicated reagents. Cells were washed with PBS after drug treatment. After centrifugation at 800 rpm for 5 minutes, the cell pellet was collected and the supernatant was discarded. The cell pellet was washed twice with PBS and fixed with 75% ethanol at 4°C for more than 4 hours. Then, the cell was re‐suspended in PBS and gathered by centrifugation at 800 rpm for another 5 minutes. The propidium iodide solution and the RNase A solution were mixed. The cells were re‐suspended by mixed solution gently and incubated at 37°C for 30 minutes in the dark. Finally, the processed cells were analysed by flow cytometry.

### 2.9 Analysis of cell senescence by β‐galactosidase staining

The senescence of HepG2 cells were determined by SA‐β‐gal staining kit (Merck Millipore). Briefly, adherent cells were incubated with PBS‐G (0.5% glutaraldehyde dissolved in PBS) for 15 minutes and washed with PBS containing 1 mmol/L MgCl_2_. The cells were stained with PBS containing 1 mmol/L MgCl_2_, 1 mg/mL X‐Gal and 5 mmol/L potassium ferricyanide for 24 hours.

### 2.10 SiRNA or plasmid transfection

LncRNA PLK4 siRNA and negative control siRNA were synthesized by GenScript. YAP CRISPR Activation Plasmid (h) (sc‐400040‐ACT) and control CRISPR Activation Plasmid (sc‐437275) were purchased from Santa Cruz Biotechnology. According to the manufacturer's procedure, HepG2 cells were transfected with Lipofectamine 2000 reagent (Life Technologies).

### 2.11 RNA isolation and real‐time PCR

Total RNA in HepG2 cells was extracted by Trizol reagent (Sigma‐Aldrich) and transcribed to cDNA by PrimeScript RT reagent kit (TaKaRa Biotechnology) according to manufacturer's protocol. Real‐time PCR was performed by the SYBR Green I fluorescent dye (TaKaRa Biotechnology), according to the manufacturer's guidelines. Each sample was assessed in triplicate experiments. The primer sequences (GenScript) used were listed in Table 1.

**TABLE 1:** Primers used for determination of mRNA expression levels in HepG2 cells

Gene	Forward sequence	Reverse sequence
p16	5′‐GGAGTTAATAGCACCTCCTCC‐3′	5′‐TTCAATCGGGGATGTCTGAGG‐3′
p21	5’‐GTCAGTTCCTTGTGGAGCCG‐3’	5’‐GAAGGTAGAGCTTGGGCAGG‐3’
Hmga1	5’‐AGGAGCAGTGACCCATGCGT‐3’	5’‐TGATGGTGGGCCTGGGGAAG‐3’
LncRNA‐1	5’‐TTAGAGAGCCGAGCCTGAT‐3’	5’‐GTTCTCCGGATTAGCTTCCTTC‐3’
LncRNA‐2	5’‐GCAGCCTCCTCCTCTTTATTC‐3’	5’‐AATGGGTGGGTACAAGTCTTAC‐3’
LncRNA‐3	5’‐CCCATCATTTACAAGCCCAAAC‐3’	5’‐TTTCAGTTCCACTCTGTCCATC‐3’
LncRNA‐4	5’‐CCGTTCCCTTGATGTCTACAA‐3’	5’‐GAGGCACCCGATATATGTTCTC‐3’
GAPDH	5’‐CTTCTTTTGCGTCGCCAGCCGA‐3’	5’‐ACCAGGCGCCCAATACGACCAA‐3’

### 2.12 Western blot analyses

Cells were lysed by a RIPA lysis buffer (Sigma), and Western blot analysis was performed according to the manufacturer's instructions (Bio‐Rad).

### 2.13 Immunofluorescence analysis

HepG2 cells were seed into 24‐well plates and given corresponding reagents for 24 hours. Cells were fixed with 4% PFA for 30 minutes at room temperature. After permeabilized with PBS‐T (0.1% Triton x‐100 dissolved in PBS), cells were blocked with PBS‐B (4% BSA dissolved in PBS) and stained with primary antibody overnight at 4°C. After that, cells were incubated with FITC‐labelled Goat Anti‐Rabbit IgG for 2 hours. 4′, 6‐Diamidino‐2‐phenylindole was used for staining the nucleus in dark for 5 minutes, and the fluorescence was observed with fluorescence microscope (Nikon).

Tumour tissues were cut into 5 µm in thickness and blocked with 1% bovine serum albumin after deparaffinization. Tumour tissues were incubated with corresponding antibodies overnight at 4°C. Secondary antibodies then incubated with the sections for 1 hour at room temperature. Fluorescence microscope (Nikon) was used to observe the fluorescence of sections.

### 2.14 Luciferase reporter assay

The luciferase reporter plasmid YAP (pGL3‐GFP‐YAP‐luc) was synthesized by GenScript. HepG2 cells were transiently transfected with the reporter plasmid in the presence of either talazoparib and/or lncRNA PLK4 siRNA. Renilla luciferase reporter (pRL‐TK; Promega) was used to detect the transfection efficiency. Luciferase activities were measured using a Dual‐Luciferase Reporter Assay System (Promega), according to the manufacturer's instructions.

### 2.15 Statistical analysis

Results were presented as mean ± SD, and the differences between groups were analysed using GraphPad Prism 5.0. The statistical analysis was determined by Student's *t* test (two group) or one‐way analysis of variance with the Student‐Newman‐Keuls test (more than two groups). *P* < .05 was considered a significance.

## 3 RESULTS

### 3.1 LncRNA PLK4 is down‐regulated in hepatocellular carcinoma

**FIGURE 1:**
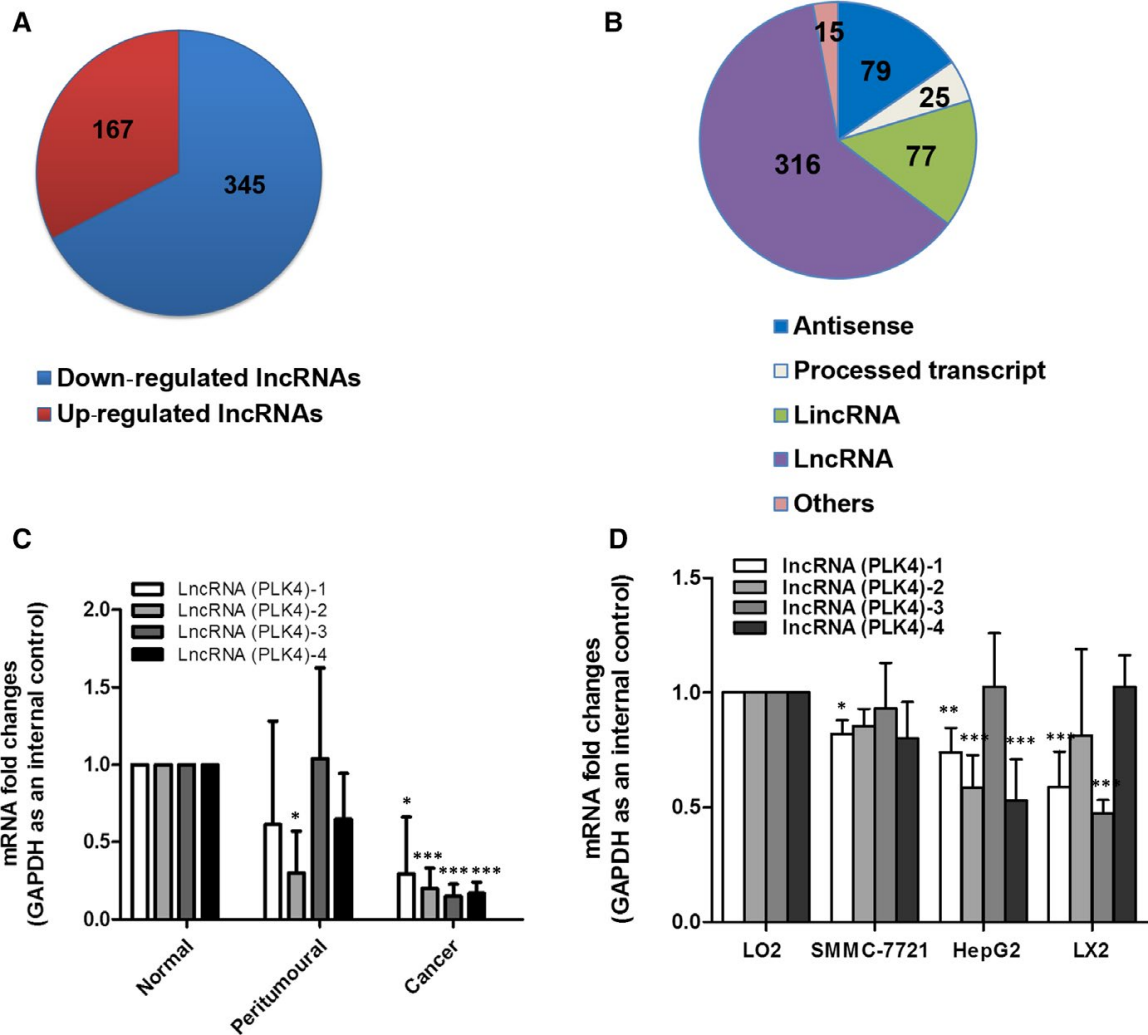
Aberrant expression of lncRNA PLK4 in HCC. Microarray analysis for lncRNA was performed with RNA extracted from normal liver tissues and patient tumour tissues with HCC. A, Pie chart representation of the number of dysregulated non‐coding RNAs during HCC tissues. (Fold changes >2; *P* < .05). B, Diagrammatic representation of the different classes of lncRNAs dysregulated during HCC. C‐D, The expression of lncRNA PLK4 was analysed by qRT‐PCR in HCC tissues and cells. Data are expressed as mean ± SD (n = 3); *P < .05 vs control, **P < .01 vs control and ***P < .001 vs control

LncRNAs involve in the pathogenesis of liver cancer and emerge as an important novel prognostic marker.^33^ However, the underlying molecular mechanism remains unknown. LncRNA expression profiles were dramatically altered in HCC, as previous studies reported.^10^ We also compared the lncRNA expression profiles between normal human liver and liver cancer tissues by lncRNAs microarray. A total of 167 up‐regulated lncRNAs and 345 down‐regulated lncRNAs with significantly differential expression were identified (Figure 1A). The majority of the dysregulated lncRNAs in HCC tissues corresponded to lncRNAs, antisense transcripts, long‐intergenic RNAs (lincRNAs) and processed transcripts (Figure 1B). Interestingly, compared to normal samples, one of the most significantly down‐regulated lncRNAs in liver cancer samples was lncRNA PLK4 (antisense transcripts). LncRNA PLK4 located at chromosome 4:128761353‐128765195 (Transcript ID: ENST00000565254, Figure S1), ~37 kb away from the PLK4 (an important oncogene) locus, prompting us to investigate it further. Real‐time PCR showed that the lncRNA PLK4 expression was markedly down‐regulated in the liver tumour tissues, compared with the adjacent tumour tissues (Figure 1C).Consistently, the expression of lncRNA PLK4 was also significantly reduced in HCC cell lines (Figure 1D). These results show that lncRNA PLK4 is down‐regulated in HCC tissues and cells.

### 3.2 Talazoparib inhibits HepG2 cell proliferation and cycle by up‐regulating lncRNA PLK4 expression

**FIGURE 2:**
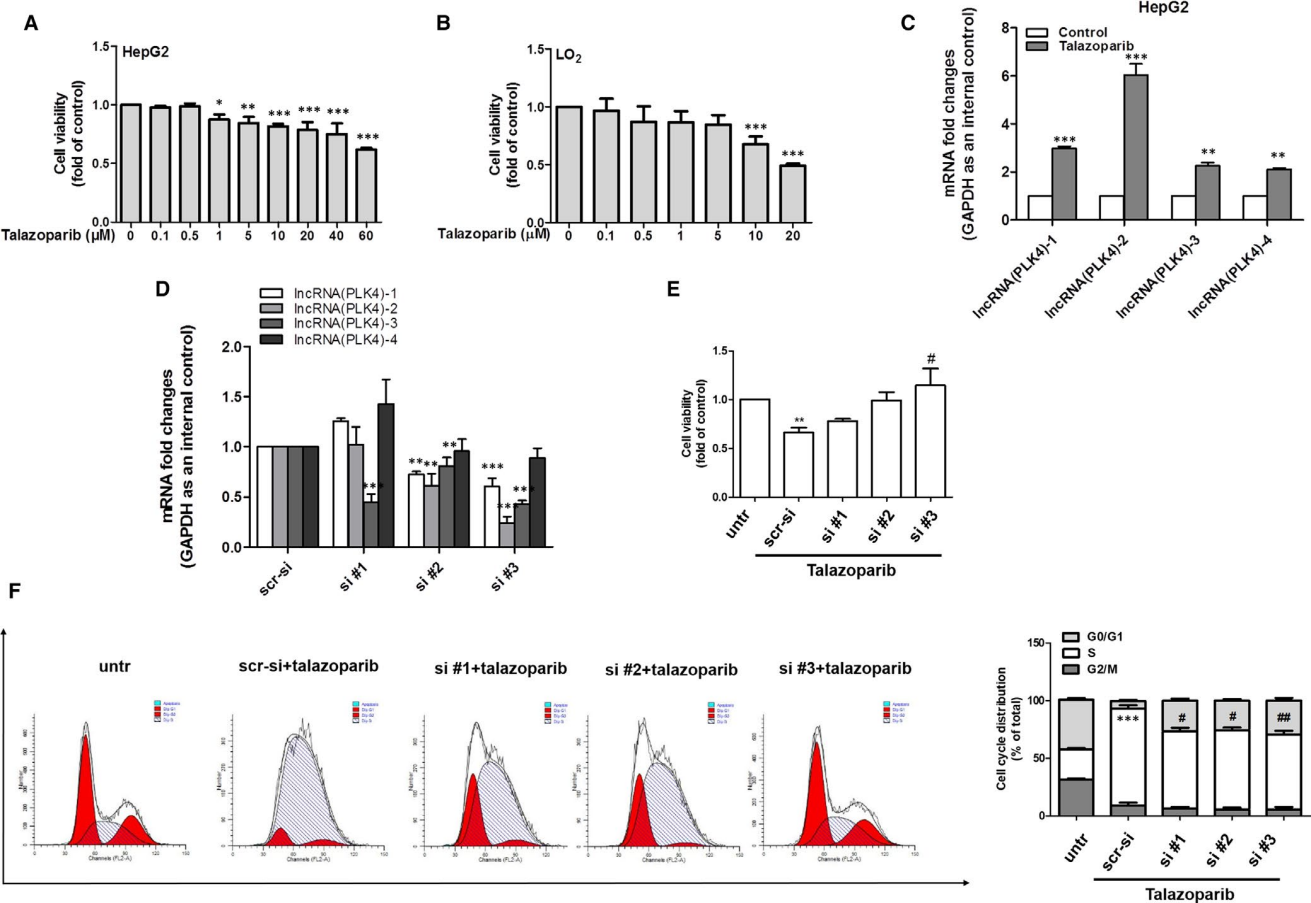
Talazoparib inhibits HepG2 cell proliferation and cycle by up‐regulating LncRNA PLK4 expression. HepG2 cells and human normal LO2 cells were treated with DMSO (0.02%, w/v) or talazoparib at 5 μmol/L concentrations for 24 h. A‐B, Cell Counting Kit‐8 analysis of the cell viability. C, Real‐time PCR analyses of lncRNA PLK4 gene. HepG2 cells were stably transfected with control siRNA or lncRNA PLK4 siRNA construction for 6 h and then treated with 5 μmol/L concentration of talazoparib for 24 h. D, Real‐time PCR analysis of the transfection efficiency. E, Cell Counting Kit‐8 analysis of the cell viability. F, Cell cycle analysis by flow cytometry. Percentages of cell cycle distributions were determined. Data are expressed as mean ± SD (n = 3); *P < .05 vs DMSO, **P < .01 vs DMSO and ***P < .001 vs DMSO. ^#^ *P* < .05 vs talazoparib and ^##^ *P* < .01 vs talazoparib

The therapeutic drugs for liver cancer are scant, we tried to find effectively novel drugs for the treatment of liver cancer. We found that talazoparib, a new and highly potent PARP1/2 inhibitor for breast cancer treatment originally, could repress the growth of liver tumour cells. Cell Counting Kit‐8 assay showed that cell viability of hepatocyte remained unchanged under talazoparib (0‐5 μmol/L) treatment, whereas talazoparib obviously inhibited HepG2 cell viability at 1 μmol/L concentration (Figure 2A,B). Importantly, 5 μmol/L talazoparib could increase the expression of lncRNA PLK4 in HepG2 cells significantly (Figure 2C). Next, lncRNA PLK4 was knocked down in HepG2 cells, using three independent small interfering RNAs and we obtained a significant knockdown efficiency (Figure 2D). The inhibitory effect of talazoparib on HepG2 cell viability was significantly ameliorated using siRNA‐mediated down‐regulation of lncRNA PLK4 (Figure 2E). Furthermore, we examined the cell cycle of HepG2 cells under talazoparib treatment by flow cytometry. As shown in Figure 2F, HepG2 cells treated with talazoparib presented higher proportions of S cells than control group. However, talazoparib‐induced S cell cycle arrest was rescued by administration of lncRNA PLK4 siRNA (Figure 2F). Therefore, talazoparib‐induced lncRNA PLK4 has a critical role in suppressing HepG2 cell growth.

### 3.3 Talazoparib induces HepG2 cell senescence

**FIGURE 3:**
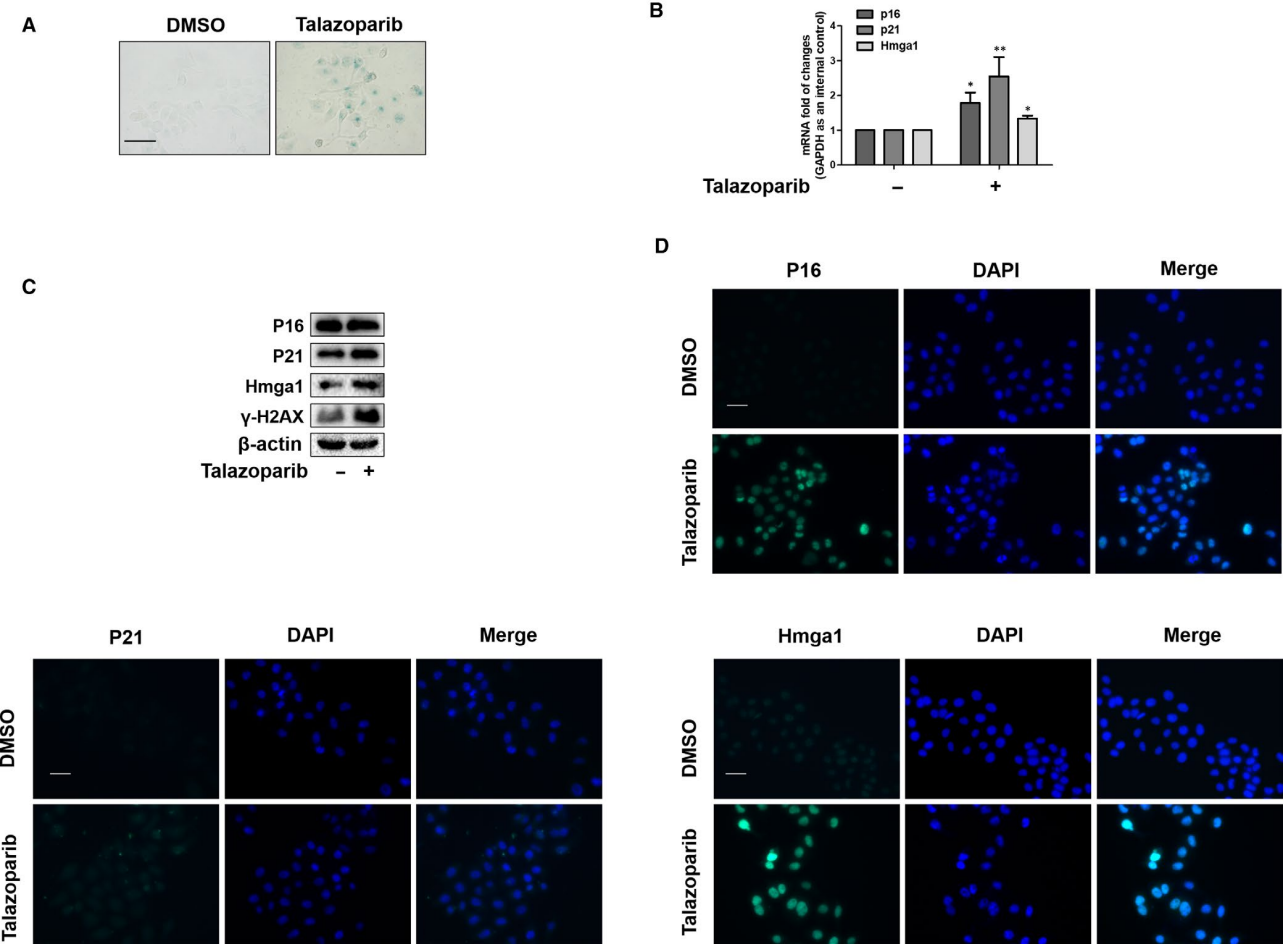
Talazoparib induces HepG2 cell senescence. HepG2 cells were treated with DMSO (0.02%, w/v) or talazoparib at 5 μmol/L concentrations for 24 h. A, Cellular senescence was assessed by β‐galactosidase staining. Scale bar, 100 µm. B, Real‐time PCR analyses of genes related to senescence, including p16, p21 and Hmga1. C, Western blot analysis of protein expression of senescence‐associated genes, including p16, p21, Hmga1 and γ‐H2AX. D, Immunofluorescence staining of p16, p21 and Hmga1. Scale bar, 50 µm. Data are expressed as mean ± SD (n = 3); *P < .05 vs DMSO, **P < .01 vs DMSO

Cellular senescence is typically characterized by cell cycle arrest, largely leading to the growth inhibition in senescent cells.^12^ Given that talazoparib could induce S cell cycle arrest of HepG2 cells, we detected the potential of talazoparib in HepG2 cell senescence. We found that SA‐β‐gal‐positive HepG2 cells increased significantly under talazoparib treatment (Figure 3A). Besides, the result of real‐time PCR demonstrated that talazoparib could promote transcription of senescence‐associated genes p16, p21 and Hmga1 (Figure 3B). Consistently, Western blotting and immunofluorescence analysis of senescence markers further indicated that talazoparib induced HepG2 cell senescence (Figure 3C,D). These results collectively suggest that talazoparib induces cell senescence in HepG2 cells.

### 3.4 Talazoparib‐induced inactivation of YAP promotes cellular senescence in HepG2 cells

**FIGURE 4:**
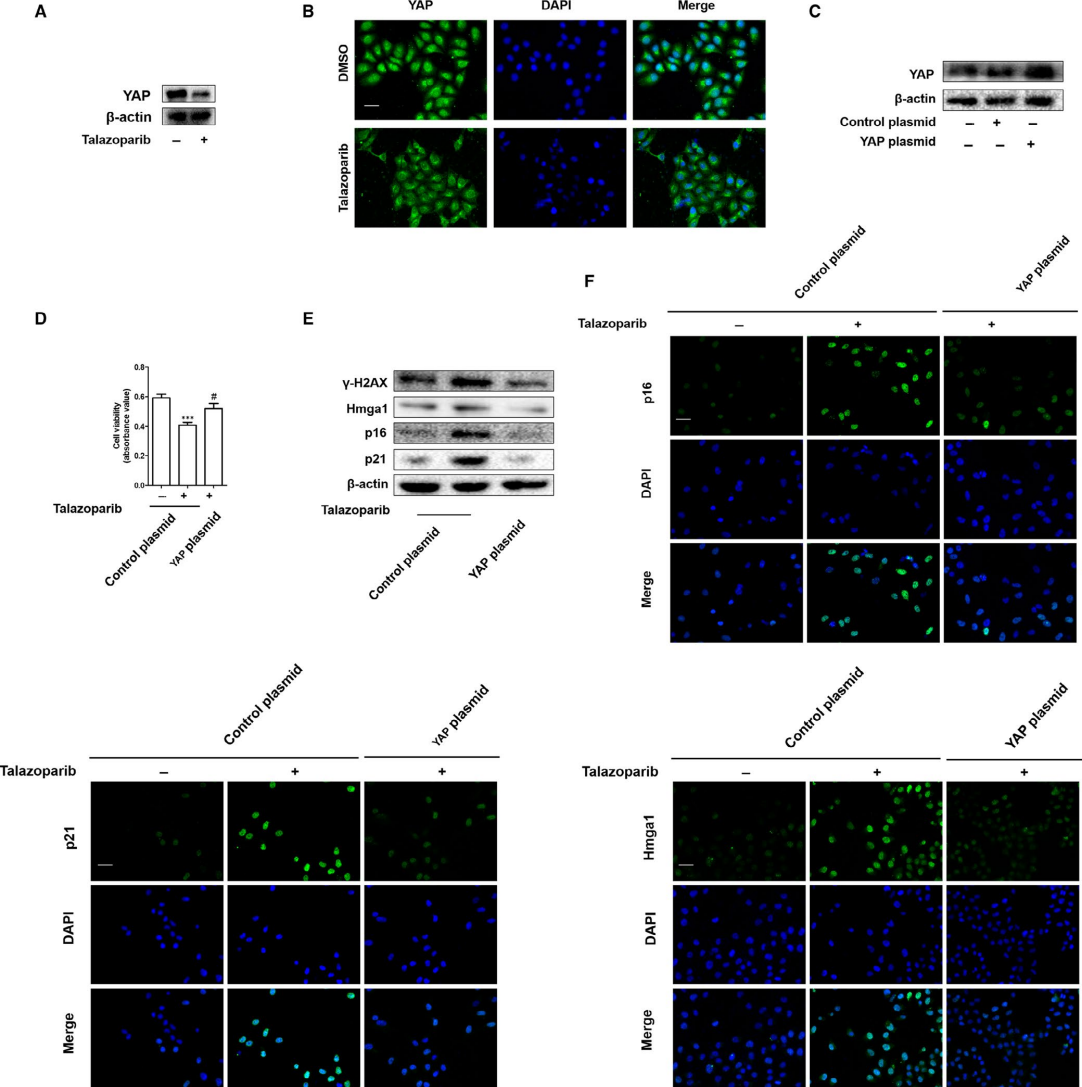
Inactivation of YAP is required for talazoparib to promote cellular senescence in HepG2 cells. HepG2 cells were treated with DMSO (0.02%, w/v) or talazoparib at 5 μmol/L concentrations for 24 h. A, Western blot analyses of YAP expression. B, Immunofluorescence staining for YAP expression. Scale bar, 50 µm. HepG2 cells were stably transfected with YAP plasmid construction for 6 h and then treated with the 5 μmol/L concentration of talazoparib for 24 h. C, Western blot analysis of the transfection efficiency. D, Cell Counting Kit‐8 was assayed for detecting the cell viability. E, Immunoblot analysis of p16, p21, Hmga1 and γ‐H2AX was performed. F, The expression of p16, p21 and Hmga1 was assessed by immunofluorescence. Scale bar, 50 µm. Data are expressed as mean ± SD (n = 3); ***P < .001 vs DMSO. ^#^ *P* < .05 vs talazoparib

The previous works including ours demonstrated that YAP was associated with cellular senescence to regulate cell growth.^18^, ^20^ Our KEGG pathway analysis also indicated that the mRNA levels of Hippo signalling pathway associated effectors were up‐regulated in HCC tissues (Figure S2). We first confirmed that whether talazoparib altered YAP translation by Western blot analysis. Talazoparib at 5 μmol/L concentration markedly down‐regulated the YAP expression (Figure 4A). Besides, immunofluorescence staining showed that talazoparib could inhibit the effect of YAP from the cytoplasm into the nucleus (Figure 4B). It is possible that YAP may involve in the talazoparib‐induced cellular senescence. Next, we explored the role of YAP in cell viability. YAP CRISPR activation plasmid significantly promoted cellular YAP expression, suggesting a high transfection efficiency (Figure 4C). Interestingly, the overexpression of YAP by transfecting YAP CRISPR activation plasmid in HepG2 cells dramatically impaired the cell viability inhibition by talazoparib (Figure 4D). Importantly, results of Western blot analysis indicated that talazoparib contributed to senescence marker induction, but declined in HepG2 cells transfected with YAP activation plasmid (Figure 4E). Consistent results were also obtained from immunofluorescence staining of p16, p21 and Hmga1 (Figure 4F). Taken together, these findings show that talazoparib promotes cellular senescence, associated with inactivation of YAP in HepG2 cells.

### 3.5 LncRNA PLK4 siRNA impairs talazoparib‐induced cellular senescence by activating YAP in HepG2 cells

**FIGURE 5:**
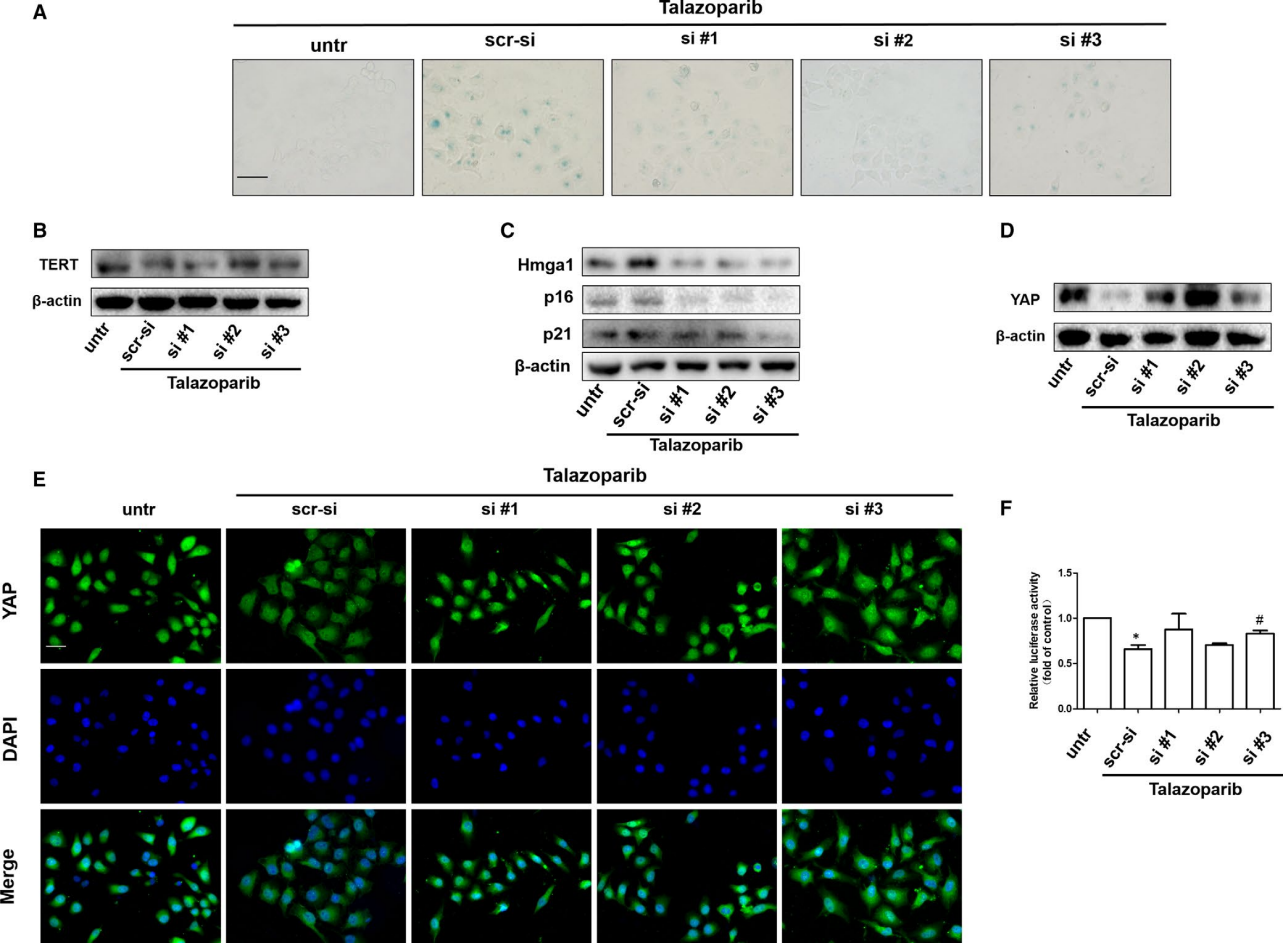
LncRNA PLK4 siRNA impairs talazoparib‐induced cellular senescence by activating YAP in HepG2 cells. HepG2 cells were stably transfected with lncRNA PLK4 siRNA plasmid construction for 6 h and then treated with 5 μmol/L concentration of talazoparib for 24 h. A, β‐galactosidase staining was performed. Scale bar, 100 µm. B‐D, Western blot analysis of TERT, p16, p21, Hmga1 and YAP expression. E, Immunofluorescence staining for YAP. Scale bar, 50 µm. F, Luciferase activities are expressed as relative units after Renilla luciferase reporter normalization. Data are expressed as mean ± SD (n = 3); *P < .05 vs DMSO. ^#^ *P* < .05 vs talazoparib

The molecular regulation networks between lncRNAs and YAP in different diseases has attracted great attention.^34^, ^35^, ^36^ Here, we assumed that lncRNA PLK4 induced by talazoparib regulated the expression of YAP, which further stimulated the transcription and translation of senescence‐associated genes. To verify this assumption, we first investigated whether lncRNA PLK4 participated in talazoparib‐induced cellular senescence in HepG2 cells. We assessed the senescence phenotype in HepG2 cells 24 hours post lncRNA PLK4 siRNA transfection. SA‐β‐gal staining showed that knockdown of lncRNA PLK4 reduced cell senescence induced by talazoparib (Figure 5A). Western blot analysis for TERT was used to assess telomerase activity. The result revealed that TERT expression was weakened by lncRNA PLK4 siRNA in talazoparib‐treated HepG2 cells (Figure 5B). Similarly, the pre‐treatment with lncRNA PLK4 siRNA obviously impaired the talazoparib‐induced senescence‐associated gene expression (Figure 5C and S3), proving that cellular senescence was lncRNA PLK4‐dependent process. Next, we investigated the effect of lncRNA PLK4 on YAP expression. As shown in Figure 5D,E, the inhibition of lncRNA PLK4 by siRNA not only could markedly weaken talazoparib inhibition in YAP expression, but also promote YAP into nucleus. Attractively, luciferase assays demonstrated that talazoparib impaired the activity of YAP, whereas lncRNA PLK4 siRNA enhanced the luciferase activity (Figure 5F). Overall, these data confirm that lncRNA PLK4 acts as the upstream mechanism of YAP inactivation to regulate talazoparib‐induced cellular senescence in HepG2 cells.

### 3.6 Talazoparib inhibits cell proliferation and promotes cell senescence in hepatocellular carcinoma xenografts

**FIGURE 6:**
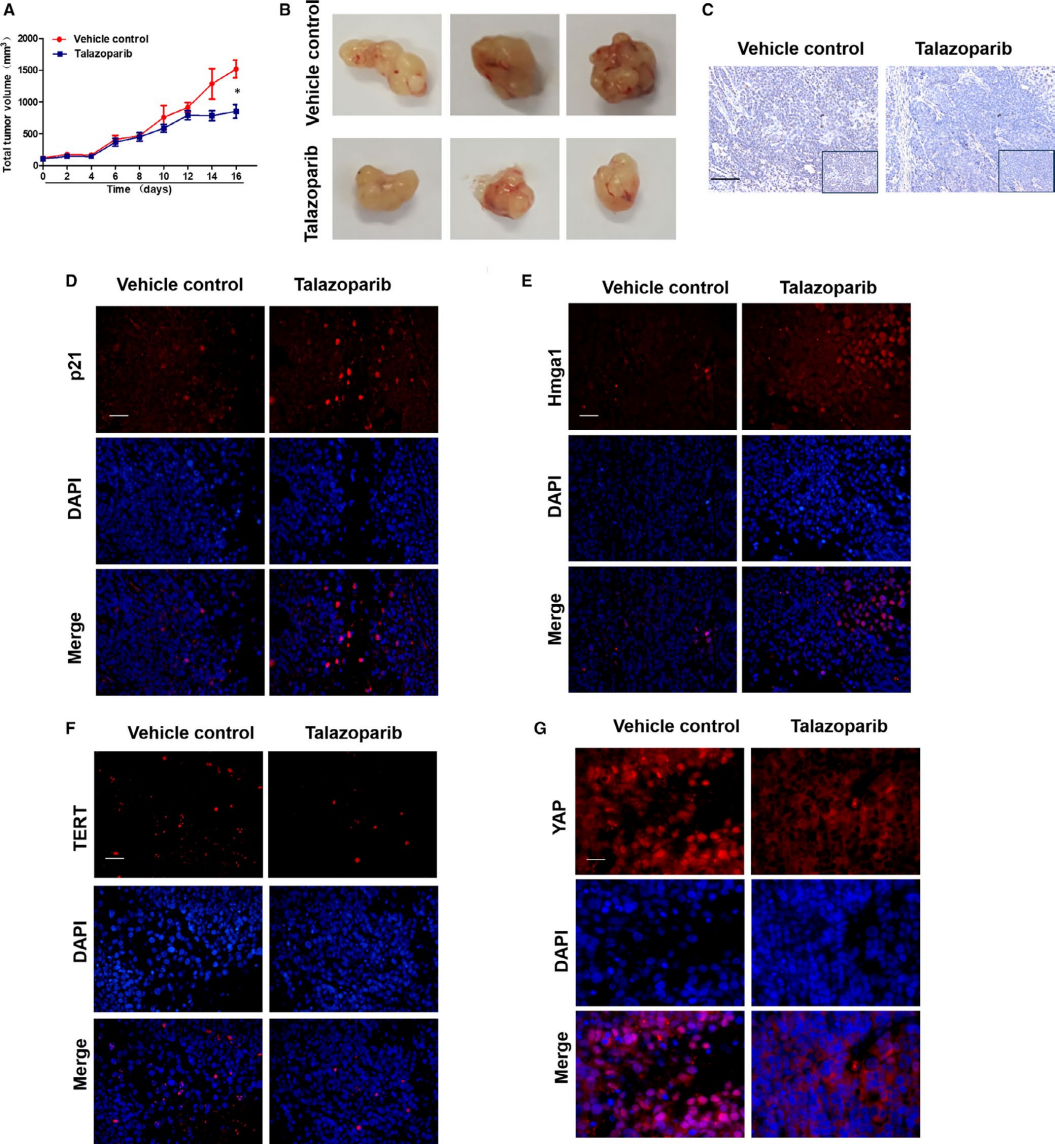
Talazoparib inhibits cell proliferation and promotes cell senescence in hepatocellular carcinoma xenografts. BALB/c nude mice were randomly divided into the following two groups (eight mice in every group): vehicle control and talazoparib. A, Quantification of tumour volume. B, Representative image of tumours dissected from vehicle‐treated control mice and talazoparib‐treated mice at day 16. C, Immunochemical staining for Ki67 of tumour tissue sections. Scale bar, 100 µm. D‐G, The expression of p21, Hmga1, TERT and YAP in tumour tissue sections was detected by immunofluorescence staining. Scale bar, 100 µm. *P < .05 vs vehicle control

To further investigate whether talazoparib could repress tumour growth in vivo, a classical xenograft model in nude mice was built through subcutaneous injection of Huh‐7 cells. We found that talazoparib treatment formed smaller tumours in mice, compared to vertical control group (Figure 6A,B). Besides, immunohistochemistry revealed that tumour cell proliferation was markedly inhibited in talazoparib‐treated mice, illustrated by decreased Ki67‐positive cells (Figure 6C). Furthermore, the result of immunofluorescence showed that talazoparib could increase the expression of p21 and Hmga1 (Figure 6D,E), reduce telomerase activity conversely in tumour tissue (Figure 6F), suggesting that talazoparib promote tumour cell senescence. Importantly, the indicated dosage of talazoparib did not cause damage to organs, including heart, liver, spleen, lung and kidney (Figure S4). In addition, we showed that YAP expression was significantly decreased in tumour tissue under talazoparib treatment (Figure 6G). Therefore, our data demonstrate that talazoparib inhibits tumour growth by repressing cell proliferation and inducing cellular senescence in HCC, and talazoparib‐induced YAP inactivation may involve in this progress.

## 4 DISCUSSION

Hepatocellular carcinoma is fundamentally a genetic disease, where numerous alterations in DNA, RNA and proteins involve in the tumour process.^37^ Researchers are devoting themselves to exploring pharmacological induction of tumour cell death, such as apoptosis,^38^ senescence,^15^ autophagy^39^ and so on, in order to seek effective strategies to treat HCC. In this study, we first identify the anti‐tumour effect of talazoparib. Importantly, we demonstrate that talazoparib‐induced lncRNA PLK4 could inhibit YAP signalling to further promote HepG2 cell senescence, which is the vital molecular mechanism that drug suppresses tumour cell proliferation.

LncRNA regulates the splicing and stability of mRNA, as well as protein translation.^40^ Recent research has improved our understanding of lncRNA function in liver diseases,^41^, ^42^ particularly in HCC.^43^ Several specific lncRNA genes could function as biomarkers of HCC diagnosis and prognosis, as well as therapeutic intervention targets. Huang et al reported that lncRNA CDKN2B antisense RNA 1 (CDKN2B‐AS1) positively regulated tumour growth and microvascular invasion, representing high tumour grade and reduced survival of HCC patients. Mechanismly, CDKN2B‐AS1 activated NAP1L1‐mediated PI3K/AKT/mTOR signalling by acting as a molecular sponge of let‐7c‐5p, affecting HCC progress.^44^ Malakar et al found that lncRNA metastasis‐associated lung adenocarcinoma transcript 1 (MALAT1) up‐regulated the expression of glycolytic genes and down‐regulated gluconeogenic enzymes by enhancing the translation of the metabolic transcription factor TCF7L2, which acts as a proto‐oncogene in HCC.^45^ Wang et al showed that lnc‐UCID (lncRNA up‐regulating CDK6 by interacting with DHX9) could competitively bind DHX9 and sequester DHX9 from CDK6‐3'UTR to increase CDK6 expression and promote HCC growth.^46^ Herein, we used lncRNA microarray to demonstrate that lncRNA PLK4 expression was significantly decreased in HCC biopsies, compared with normal group (Figure 1A,B). Real‐time PCR analysis also verified the results (Figure 1C). The decreased lncRNA PLK4 was identified in both SMMC7721 and HepG2 cells, as well as human HSC‐LX2 cells (Figure 1D). Our results indicate that lncRNA PLK4 may be a promising marker and intervention target for HCC.

Talazoparib, a novel highly potent PARP1/2 inhibitor, has advantageous on metabolic stability, oral bioavailability and pharmacokinetic properties. Talazoparib could inhibit tumour cell proliferation and metastasis, especially of breast cancer and small cell lung cancer (SCLC).^47^, ^48^ Talazoparib is recently in multiple phase II and phase III clinical trial and represents a promising anti‐tumour drug.^49^ However, there are no studies on assessing the effect of talazoparib on HCC. Herein, we investigated the role of talazoparib in HCC. Huh‐7 cells were subcutaneously injected in nude mice to form tumour xenograft, as previous reported. We found that talazoparib could not only impair HepG2 cell viability in vitro (Figure 2A), but also delay tumour growth and inhibit tumour cell proliferation significantly (Figure 6A‐C). Interestingly, talazoparib dramatically up‐regulated lncRNA PLK4 mRNA level (Figure 2C), whereas cell viability was resumed when lncRNA PLK4 was genetically reduced by siRNA (Figure 2E). Besides, talazoparib‐induced S cell cycle arrest, and lncRNA PLK4 siRNA significantly impaired the effect. We assumed that talazoparib might trigger tumour cell senescence (Figure 2F). As expected, talazoparib induced the senescence of HepG2 cells, illustrated by the accumulation of SA‐β‐gal staining positive cells (Figure 3A) and the increased expression of p16, p21 and Hmga1(Figure 3A‐C).

Recently, roles between lncRNA and cell senescence have been recognized in tumorigenesis. How these two seemingly separate functions are intertwined that remains unclear. Hippo kinase signalling is inactivated in many cancers, playing important role in tumour progression and invasion,^50^, ^51^, ^52^ including HCC.^53^ A previous study confirmed that the key molecule of Hippo kinase signalling, YAP, increased resistance to RAF‐ and MEK‐targeted cancer therapies.^54^ Sun et al reported that the mammalian homolog of Usp7, HAUSP, could function as a vital therapeutic target for HCC by modulating YAP ubiquitination and degradation.^55^ Chen et al found that high mobility group box 1 (HMGB1) bound to GA‐binding protein alpha and promoted YAP expression, contributing to liver tumorigenesis by inducing hypoxia‐inducible factor 1 alpha (HIF1‐α)‐dependent aerobic glycolysis.^56^ In this study, we demonstrated that talazoparib repressed the translation of YAP (Figure 4A). Besides, numerous YAP could translocate from the nucleus to the cytoplasm under the treatment of talazoparib (Figure 4B). Moreover, talazoparib‐induced inhibition of cellular viability was resumed by YAP overexpression plasmid (Figure 4D). Furthermore, YAP activation could weaken the pro‐senescent response of talazoparib in HepG2 cells, illustrated by decreased SA‐β‐gal‐positive cells and the expression of p16, p21, Hmga1 and γ‐H2AX (Figure 4E,F). In addition, our experiments in vivo demonstrated that talazoparib promoted tumour cell senescence and reduced YAP expression conversely (Figure 6D‐G). These discoveries collectively suggest that talazoparib‐induced cell senescence by inhibiting YAP could repress tumour cell growth, consistent with the previous study that increased YAP delayed cellular senescence.^28^, ^29^, ^30^ LncRNA up‐regulation has been gradually recognized as the mediator of promoting a strong senescence phenotype, inhibiting cell growth.^57^ We also identified that lncRNA PLK4 inhibition with siRNA markedly suppressed talazoparib‐induced pro‐senescent response, as reduced by SA‐β‐gal‐positive cells (Figure 5A) and the expression of p16, p21 and Hmga1 (Figure 5C). It is well known that the reduction of telomerase activity and telomere length represents cell senescence.^14^ Our results demonstrated that talazoparib induced a decrease of telomerase activity, while lncRNA PLK4 siRNA significantly impaired the effect (Figure 5B). Attractively, the inhibition of lncRNA PLK4 by siRNA increased YAP expression markedly (Figure 5D,E). Luciferase assays indicated that talazoparib impaired the activity of YAP, whereas lncRNA PLK4 siRNA enhanced the luciferase activity (Figure 5F). Thus, our data show that YAP inactivation serves as a downstream event of lncRNA PLK4 down‐regulation, and triggers tumour cell senescence, which might be the key mechanism that talazoparib inhibits hepatoma cell growth. It is urgent to further determine the underlying mechanism of lncRNA PLK4 regulation in YAP inactivation in HCC cells.

**FIGURE 7:**
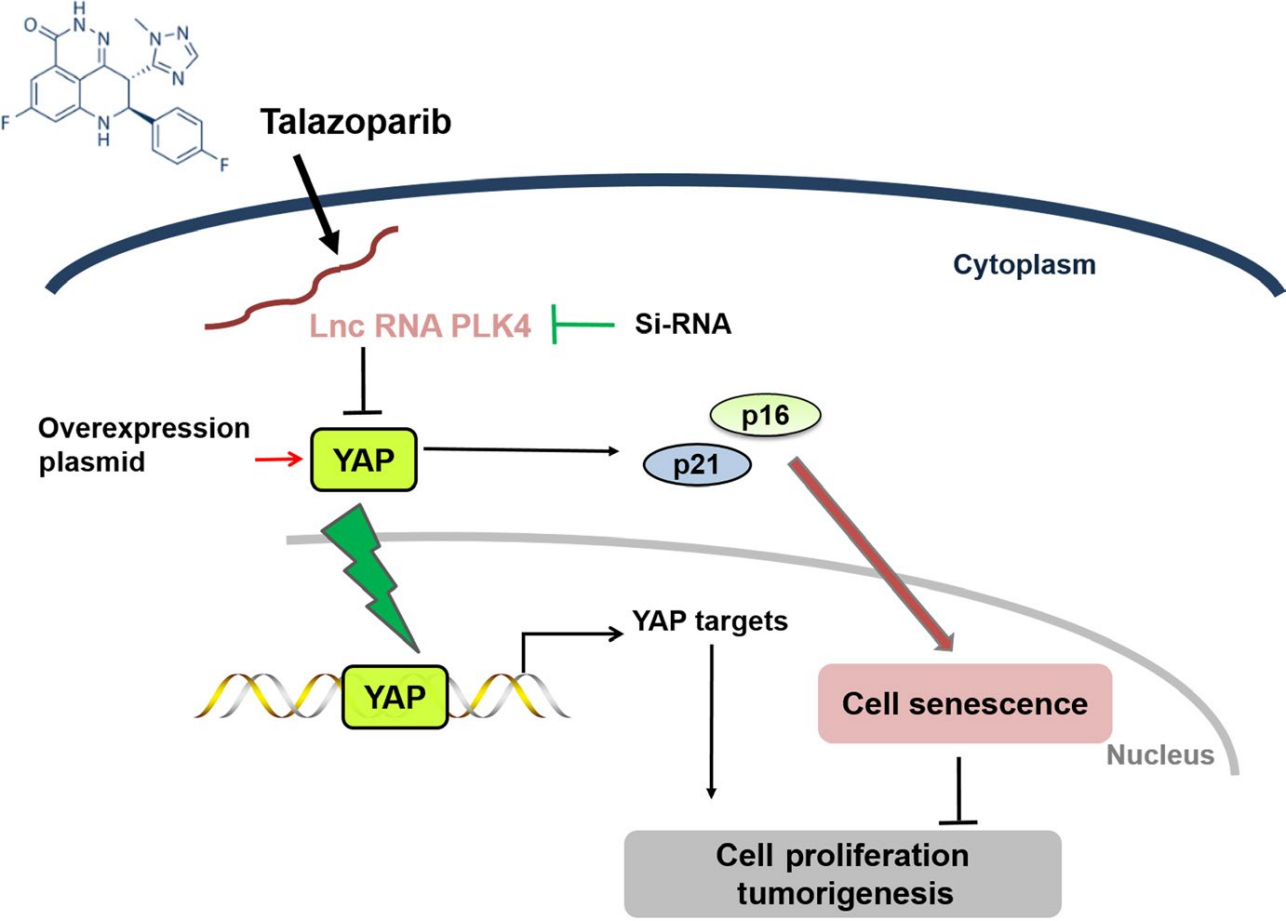
Schematic diagram illustrating that talazoparib modulated lncRNA PLK4/YAP signalling axis to regulate cell senescence in HepG2 cells and inhibited tumour cell growth

In summary, our findings demonstrate that talazoparib has an anti‐cancer effect in HCC xenografts. Mechanistically, talazoparib inhibits tumour cell growth through promoting lncRNA PLK4 up‐regulation specifically, followed by YAP inactivation, ultimately leading to cellular senescence of hepatoma cells. The senescence of tumour cells induced by talazoparib may prove to be a potential strategy for the treatment of HCC (Figure 7).

## CONFLICT OF INTEREST

The authors declare no conflict of interest.

## AUTHOR CONTRIBUTIONS

JY, JHH and GLY performed the experiments and analysed data. Y.X, D.H and WFX are responsible for the collection of materials. J.Y and ZSS drafted the work. CAP, Z.F and SJJ revised the intellectual content of manuscript. SZT corrected some English and format mistakes. WSJ provided materials and technique supports. All authors have read and approved the final manuscript.

## Supporting information

Fig S1‐S4

## Data Availability

All data generated or analysed during this study are included in this published article.
