# Supplementary material for: A novel lncRNA PLK4 up‐regulated by talazoparib represses hepatocellular carcinoma progression by promoting YAP‐mediated cell senescence
Source: J Cell Mol Med. 2020 Apr 3;24(9):5304–16. doi: 10.1111/jcmm.15186 (PMC7205816; doi:10.1111/jcmm.15186)
Supplement: Supplementary file 1 — Fig S1‐S4 [file JCMM-24-5304-s001.docx]

**
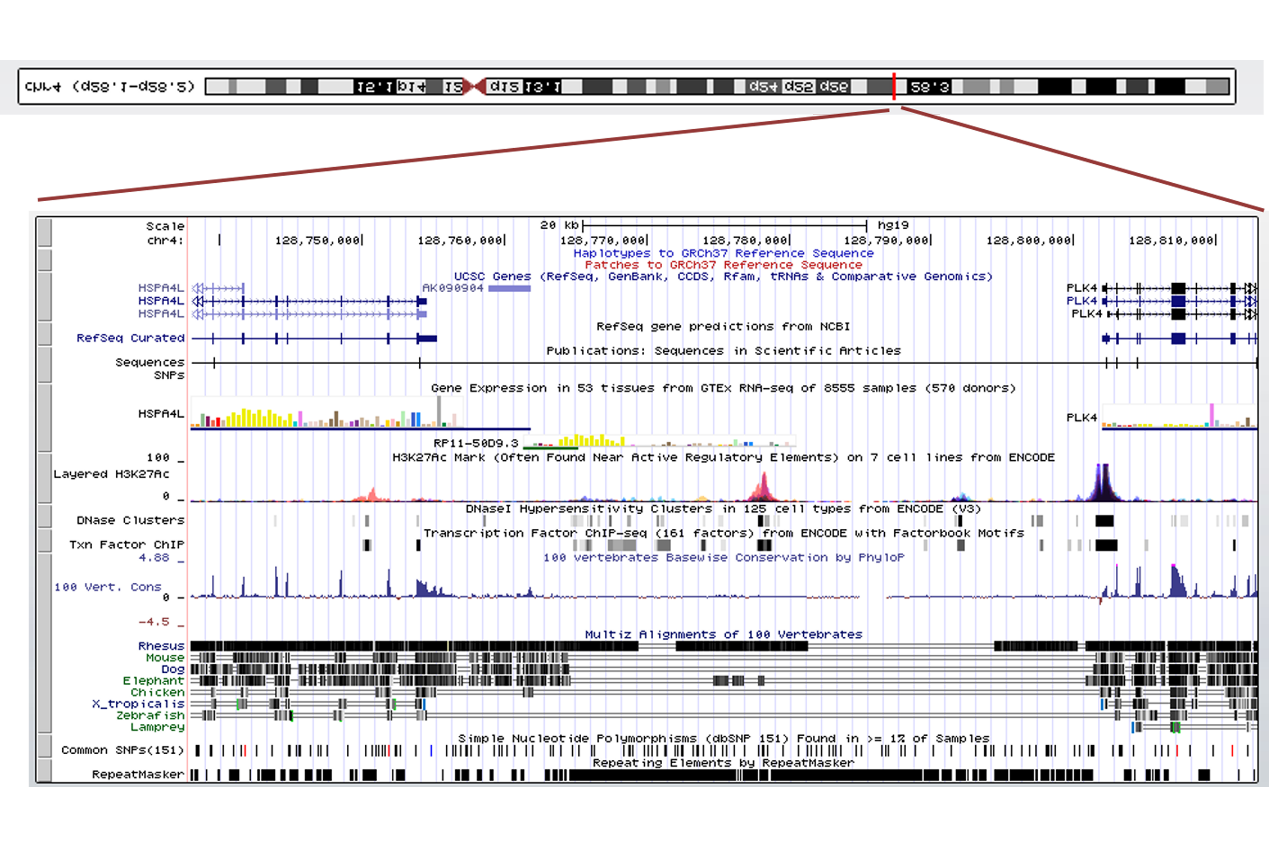
Supplementary Fig. 1 Transcript of lncRNA PLK4.**

**
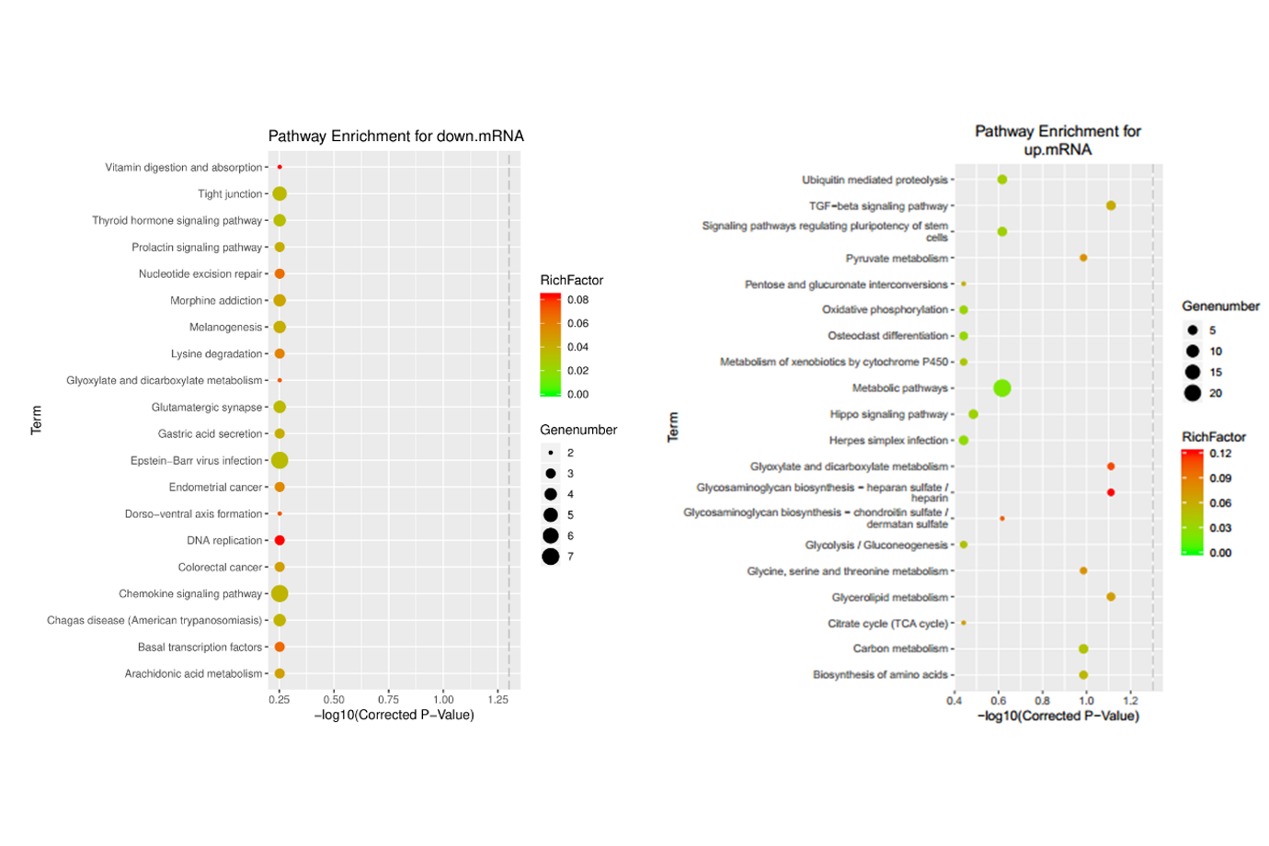
**

**Supplementary Fig. 2 KEGG pathway analysis.**

**
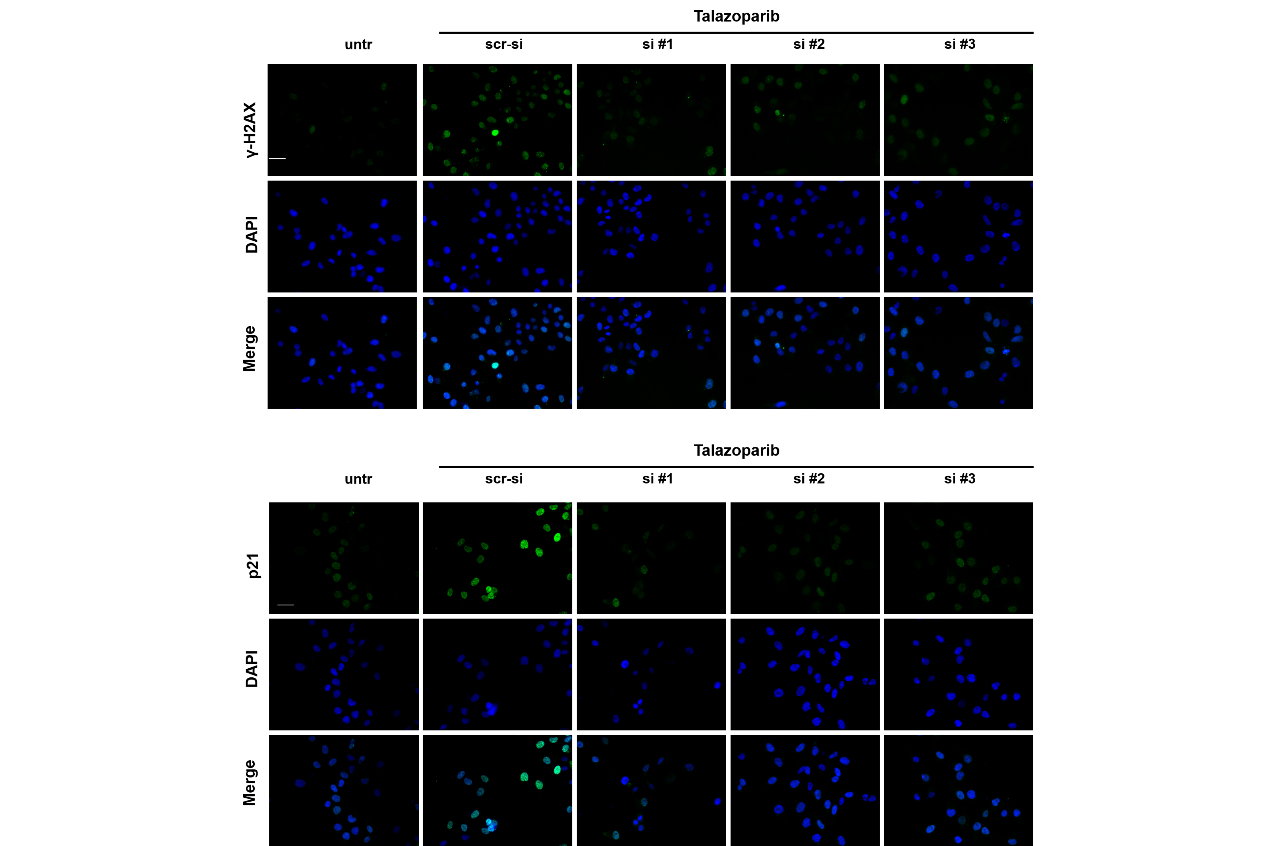
**

**Supplementary Fig. 3 LncRNA PLK4 siRNA impairs talazoparib-induced cellular senescence.** Immunofluorescence staining for p21 andγ-H2AX. Scale bar, 50 µm.

**
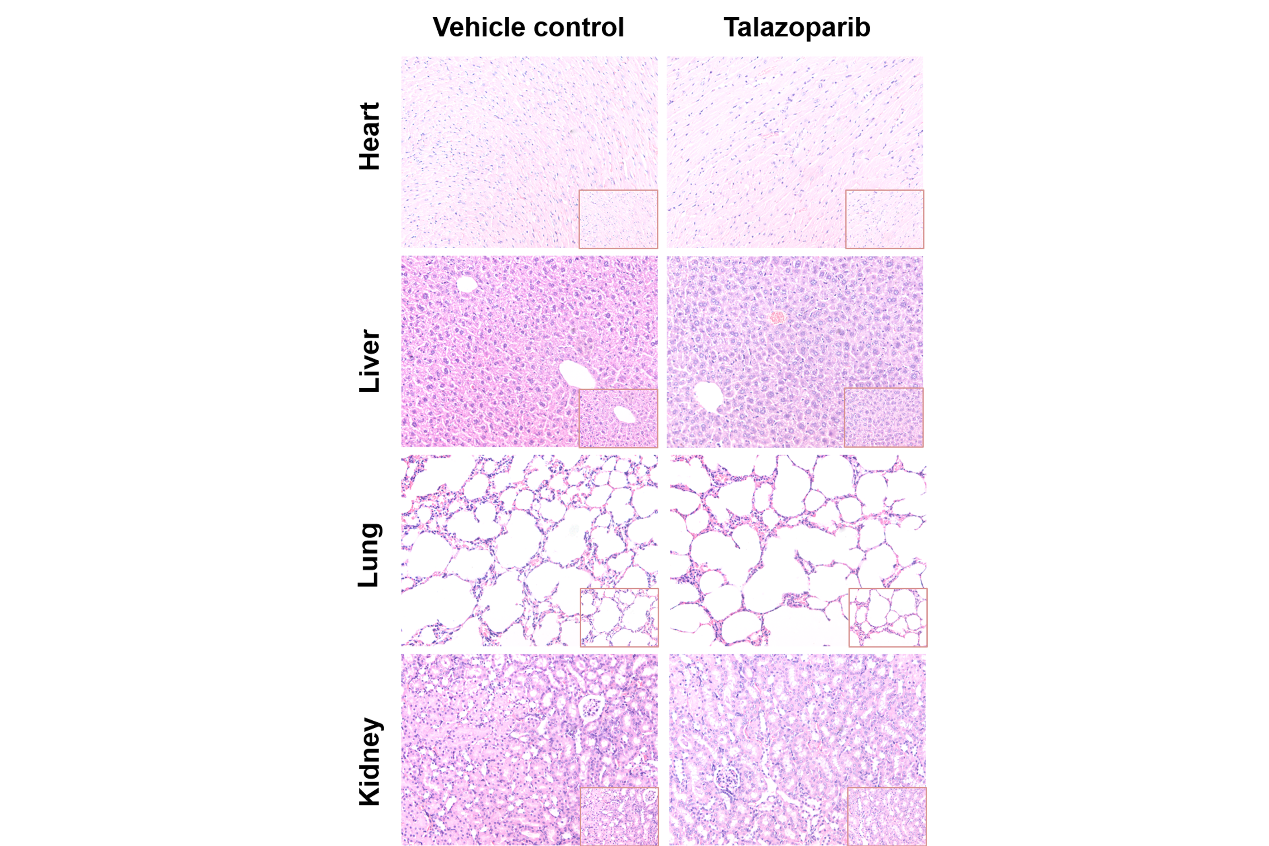
**

**Supplementary Fig.4 Talazoparib did not cause damage to important organs.** Representative microphotograph of H&E-stained sections, including heart, liver, spleen, lung, kidney. Scale bar, 100 µm.
